# Whole-Genome Sequence of Aneurinibacillus migulanus TP115, a Potential Fish Probiotic Isolated from Oreochromis niloticus Gut

**DOI:** 10.1128/mra.00861-22

**Published:** 2022-10-27

**Authors:** Sulav Indra Paul, Ashikur Rahman, M. Mahbubur Rahman

**Affiliations:** a Institute of Biotechnology and Genetic Engineering, Bangabandhu Sheikh Mujibur Rahman Agricultural University, Gazipur, Bangladesh; Montana State University

## Abstract

We report the whole-genome sequence of a promising fish probiotic, Aneurinibacillus migulanus TP115, which was isolated from the gut of Nile tilapia (Oreochromis niloticus). The *de novo* assembly resulted in an estimated chromosome size of 5,556,554 bp, with 5,576 open reading frames.

## ANNOUNCEMENT

Fish probiotics are particularly important for growth promotion and disease management in aquaculture (1). Aneurinibacillus migulanus is a prospective biocontrol agent against bacterial pathogens because of its ability to produce various antimicrobial compounds (2). The presented genome information for A. migulanus TP115 will facilitate the understanding of its safety and probiotic potential for safe use in aquaculture.

We isolated A. migulanus TP115 in 2018 from a healthy Nile tilapia in Bangladesh. The abdomen of the fish was cut aseptically, and the gut was removed for the isolation of probiotic bacteria. One-gram homogenates of the intestinal segments were serially diluted and spread on de Man, Rogosa, and Sharpe (MRS) agar plates, followed by incubation at 28°C for 48 h. A single whitish-gray colony growing on the MRS plate after 48 h of incubation was identified as Aneurinibacillus migulanus (GenBank accession number MW512511.1) based on 16S rRNA gene sequence homology. The primers used for amplification were 8F (5′-AGAGTTTGATCCTGGCTCAG-3′) and 1492R (5′-GGTTACCTTGTTACGACTT-3′). TP115 exhibits potent *in vivo* growth promotion and suppresses motile *Aeromonas* septicemia in Oreochromis niloticus (3). Prior permission was obtained from the Institute of Biotechnology and Genetic Engineering (IBGE) ethical review committee for the animal experiments (approval number IBGE-ERC-008).

The genomic DNA of TP115 was extracted from the original stock in 2019. The same single colony as used for 16S rRNA sequencing was inoculated in MRS broth and incubated at 28°C for 48 h. High-quality genomic DNA was extracted using a GeneJET genomic DNA purification kit (Thermo Fisher Scientific, USA), and the quality and quantity of the DNA were checked using a NanoDrop spectrophotometer (Thermo Fisher Scientific). A paired-end DNA library was prepared using the Nextera XT library preparation kit (Illumina, San Diego, CA, USA) according to the manufacturer's instructions (4). Sequencing (600 cycles) was performed using the MiSeq benchtop sequencer (Illumina) (5, 6) and yielded a total of 3,528,592 reads (1,764,296 paired-end reads) and 762,217,704 bases. Trimmomatic v.0.38 (7) was used to remove the sequence adaptors, and quality filtering was performed using PRINSEQ v.0.20.3 (8). *De novo* assembly was performed using SPAdes v.3.9.0 (9), and QUAST v.5.0.2 (10) was used for quality assessment of the assembled genome. Genome annotation was performed using the NCBI Prokaryotic Genome Annotation Pipeline (PGAP) (https://www.ncbi.nlm.nih.gov/genome/annotation_prok) (11). Using SpeciesFinder v.2.0 (https://cge.food.dtu.dk/services/SpeciesFinder), the bacterium was identified as Aneurinibacillus migulanus (12). Default parameters were used for all software unless otherwise specified.

The chromosome size of TP115 is 5,556,554 bp distributed in 113 contigs, with a G+C content of 43.7%, genome coverage of 137×, and no plasmids. The genome contains 5,576 total coding sequences (CDSs), with 5,405 CDSs encoding putative proteins. The *N*_50_ and *L*_50_ values of the assembly were 126,253 bp and 12, respectively. The largest and smallest contigs were 567,280 bp and 505 bp, respectively. PGAP predicted 134 RNA genes (108 tRNA genes, 20 rRNA operons, and 6 noncoding RNA [ncRNA] genes) and 171 pseudogenes. RAST v.2.0 (13) predicted 316 subsystems and 2,090 protein-coding genes putatively assigned to functional categories. Using antiSMASH v.6.1.1, a total of 13 gene clusters for secondary metabolites were predicted for the genome of the A. migulanus TP115 (14). β-Lactones, cyclic lactone autoinducers, terpenes, siderophores, ranthipeptides, proteusins, nonribosomal peptides, polyketides, arylpolyenes, sactipeptides, and lasso peptides are among the predicted secondary metabolites.

### Data availability.

The whole-genome shotgun project for Aneurinibacillus migulanus TP115 has been deposited in GenBank under the accession number JAMZFU000000000. The version described in this paper is version JAMZFU010000000. The raw reads and raw sequencing data are available under SRA accession number SRX15894335, BioProject accession number PRJNA851951, and BioSample accession number SAMN29257231.
